# The Relationship between Levels of PCBs and Pesticides in Human Hair and Blood: Preliminary Results

**DOI:** 10.1289/ehp.6916

**Published:** 2004-05-27

**Authors:** Larisa Altshul, Adrian Covaci, Russ Hauser

**Affiliations:** ^1^Department of Environmental Health, Exposure, Epidemiology and Risk Program, Harvard School of Public Health, Boston, Massachusetts, USA; ^2^Toxicological Center, University of Antwerp, Wilrijk, Belgium; ^3^Department of Environmental Health, Occupational Health Program, Harvard School of Public Health, Boston, Massachusetts, USA; ^4^Vincent Memorial Obstetrics and Gynecology Service, Andrology Laboratory and In Vitro Fertilization Unit, Massachusetts General Hospital, Boston, Massachusetts, USA

**Keywords:** exogenous and endogenous contaminants, organochlorines, pesticides, polychlorinated biphenyls (PCBs)

## Abstract

Human hair as a biologic measure of exposure to persistent organic pollutants (POPs) has some advantages over the more commonly used blood and adipose tissue samples. However, one of the primary limitations is the difficulty in distinguishing between exogenous and endogenous contamination. In addition, there are currently no standardized methods for hair sample collection, washing, and chemical analysis. There is also very limited information describing the correlation between levels of organic contaminants in hair and other body compartments. To explore levels of POPs in blood and hair, samples from 10 volunteers were collected and analyzed for select organochlorine pesticides and 57 individual polychlorinated biphenyl (PCB) congeners. We demonstrated that the method for analyzing organic contaminants in human hair was reliable and reproducible. Washing hair with shampoo decreased levels of PCBs, pesticides, and lipids by 25–33% on average and up to 62% for low-chlorinated congeners. The percentage of lipids and the levels of organochlorines in hair were higher than in serum. We found strong correlation (*r* = 0.8) between *p*,*p*′-DDE (dichlorodiphenyldichloroethylene) levels in hair and blood and moderate correlations for the more persistent PCB congeners, but no correlations or weak correlations for other organochlorines. The present study provides preliminary evidence on the utility of hair analysis for POPs; however, further larger studies are recommended before hair analysis can be successfully applied in epidemiologic studies on POPs.

There is an extensive scientific literature describing the levels of organochlorines in human tissues, mainly in blood (Bush et al. 1984; Luotamo et al. 1985), including cord blood (Covaci et al. 2002b; Korrick et al. 2000), breast milk (Johansen et al. 1994; Mes et al. 1987), and adipose tissues (Covaci et al. 2002a; Focardi et al. 1986). Human hair is not commonly used as a biologic measure of exposure to persistent organic pollutants (POPs), although it has some advantages over the more commonly used blood and adipose tissue samples. Blood is not always available in sufficient amounts for a reliable analysis, whereas tissues need to be obtained by more invasive procedures or at surgery or autopsy. Because the collection of hair is simple, inexpensive, and noninvasive, subject compliance is high and hair can easily be collected from both adults and children. Hair sampling would not require the same precautions and conditions for handling, storage, and shipment as does blood, milk, or tissues. Hair sample collection would be especially useful in large epidemiologic studies where hair samples can be remotely collected by subjects and mailed to the investigator. In addition, studies with small children would be more feasible because the collection of blood limits participation rates. Finally, levels of environmental chemicals in hair are relatively stable (Bencze 1990a, 1990b) and may occasionally be high.

However, there are several limitations that need to be overcome before hair can be widely used as a matrix for biologic monitoring. One of the primary limitations is the difficulty in distinguishing between exogenous and endogenous contamination. In addition, there are currently no standardized methods for hair sample collection, washing, and chemical analysis. Another limitation is the lack of information regarding the rate of elimination, metabolization, and distribution of organic pollutants between hair and other body matrices. Finally, when exposure levels are low, as in general population studies, the sensitivity of the analytical methods for hair analysis might be a potential limitation.

There is a significant literature describing methods for analyzing hair for metals, specifically methyl mercury and arsenic (Jacobs 1996; Tsalev 1995), abused and therapeutic drugs (Beumer et al. 2001; Nakahara 1999), and anabolic steroids (Kicman and Gower 2003). These analyses have been performed for many years, and accordingly, analytical methods have been optimized and validated. Moreover, for some metals, reference values for levels found in the general population are available (Seifert et al. 2000). However, unlike metals, methods for hair analysis to assess exposure to polychlorinated biphenyls (PCBs), organochlorine pesticides (OCPs), and other POPs are not fully developed and validated.

Previous studies describing measurement methods for POPs in hair are limited. Schramm and colleagues (Schramm 1997; Schramm et al. 1992) were among the first scientists to recognize the potential utility of hair analysis for the assessment of human exposure to polychlorinated dibenzodioxins (PCDDs) and polychlorinated dibenzofurans (PCDFs). Subsequently, simplified analytical methods for the extraction of PCBs, dichlorodiphenyltrichloroethane (DDT) and hexachlorocyclohexane (HCH) isomers from human hair were developed by Dauberschmidt and Wennig (1998) and by Covaci and Schepens (2001). Covaci and Schepens (2001) explored the relationship between select PCBs and OCPs in hair and breast milk from one individual and found that lipid-adjusted levels were comparable for most of compounds. Recently, Nakao et al. (2002) measured levels of PCDDs/PCDFs and coplanar PCBs in hair and blood collected from six healthy donors. They found moderate correlations between levels of 1,2,3,4,7,8-hexaCDD and 2,3,4,7,8-pentaCDF in hair and blood. The hair and blood levels of the other isomers of PCDD/PCDFs were not correlated. To the best of our knowledge, there are no other data describing the correlation between levels of organic contaminants in human hair and other body compartments, such as blood or tissues.

In June 2001, the Agency for Toxic Substances and Disease Registry convened a meeting of experts to review and discuss the current state of the science on hair analysis and the feasibility of using hair analysis in assessing environmental exposure (Harkins and Susten 2003). One of the conclusions from this meeting was that for most substances, with the exception of methyl mercury, for which the levels in hair are a biomarker of exposure, hair analysis is currently not a reliable indicator of environmental exposure or internal body burden. More research is needed before hair analysis can be considered a valid tool for human environmental exposure and health studies. They also concluded that there is limited available information on the utility of hair analysis for environmentally relevant organic pollutants but that the knowledge in this field should be expanded.

The objectives of the present study were to validate analytical methods for measuring PCBs and OCPs in human hair, to evaluate the effect of hair washing on the levels of hair contaminants, to assess endogenous versus exogenous exposure, and to determine the relationship between the levels of these compounds in hair and blood. To determine the utility of hair analysis for biomonitoring, we needed to compare this new method with well-established methods and to assess the comparability of the results from serum and hair analysis.

## Materials and Methods

All subjects signed an informed consent approved by the Harvard School of Public Health Human Subjects Committee. The subjects were a convenience sample of volunteers.

### Blood and hair samples.

Blood samples were collected in red top Vacutainer tubes, and the serum fraction was removed after being separated with centrifugation. The serum was stored in solventrinsed glass vials with Teflon-lined caps at −20°C until extraction. There was a significant interval (ranging from 10 to 25 months) between collecting serum and hair samples. Because there is no standard protocol for collecting hair samples, scalp hair was collected by study participants during their routine haircut. Because men and some of the women had short hair, the hair was cut from all areas of the scalp. For women with long hair, only the distal portion of hair was cut. We did not collect information on participants’ hair color or hair treatments, such as dyeing, permanent waving, or personal care products.

### Hair washing.

Hair samples were covered with 35 mL hot water, sonicated for 30 min, and then dried with paper towel. For washing hair with shampoo, we placed hair in a 40-mL screw-cap vial, filled it with 35 mL deionized water, added one drop of mild Johnson & Johnson Baby Shampoo (Johnson & Johnson; Skillman, NJ) and vigorously shook the vial for 3 min. [The shampoo was separately tested for organochlorines, and all concentrations were < 0.02 ng/g, except that of hexachlorobenzene (HCB), which was 0.067 ± 0.004 ng/g.] The washing solution was decanted (and saved for analysis), the hair was rinsed five times with 30 mL deionized water each rinse, and the rinses were added to washing solution. For the hair washed with shampoo twice, the washing procedure was repeated by adding shampoo the second time before rinsing the hair.

### Laboratory analysis.

All samples (blood serum, hair, and washing liquid) were analyzed for 57 individual PCB congeners and chlorinated pesticides. Details of hair extraction (Covaci and Schepens 2001), serum analyses (Korrick et al. 2000), and extraction of the washing liquid [U.S. Environmental Protection Agency (EPA) 1984] have been reported and are briefly described below. Before extraction, each sample was spiked with the surrogate compounds PCB-30 and PCB-112 [International Union of Pure and Applied Chemistry (IUPAC) nomenclature; Ballschmiter et al. 1992] to monitor the efficiency of the extraction procedure. Blood serum samples were denatured with methanol and extracted three times with a 1:1 mixture of hexane and ethyl ether. The hair samples were extracted by submerging hair in 3N hydrochloric acid, incubating it overnight at 40°C, and extracting three times with a mixture of *n*-hexane and dichloromethane (4:1, vol/vol). Water samples were extracted three times with dichloromethane. All solvent extracts were dried with anhydrous sodium sulfate, concentrated using a Kuderna-Danish evaporator followed by concentration under the stream of purified nitrogen. The percentage of lipids in hair and serum were determined gravimetrically by weighing an aliquot of the extract (20%). The remaining extract was concentrated to approximately 1–2 mL in a Kuderna-Danish apparatus followed by nitrogen evaporation.

For all three matrices, the extract was cleaned up using a chromatographic column packed with anhydrous sodium sulfate, 3% deactivated silica gel, and 2% deactivated aluminum oxide and eluted with 20 mL hexane. The sample extracts were concentrated to 100 μL, analyzed by dual capillary high resolution gas chromatography with electron capture detection and quantified based on the response factor of each analyte relative to the internal standard (PCB-166), added before gas chromatography injection. The average values obtained from both columns were reported for each target analyte unless the difference between two results exceeded 20%, in which case the lower value was reported. PCB concentrations were reported as individual congeners and as the sum of all congeners assayed (∑PCB). All final concentrations were reported after subtracting the amount of the analyte measured in the procedural blank.

### Statistical analysis.

For data analysis, we used SAS, Version 8.2 (SAS Institute Inc., Cary, NC). Descriptive analyses of subject characteristics were performed. We used Spearman correlation coefficients to determine correlations between hair and blood levels of organochlorines.

## Results

Ten Caucasian adults (five men and five women) participated. The men’s ages ranged from 25 to 43 years, with a mean ± SD of 34 ± 7.6 years. The women’s ages ranged from 39 to 53 years, with a mean ± SD of 43 ± 7.3 years. None of the subjects reported occupational exposure to PCBs or pesticides.

The average recoveries ± SD for two surrogates, PCB-30 and PCB-112, added to hair samples were 73 ± 5% and 82 ± 7% and for washing liquid they were 86 ± 18% and 86 ± 20%, respectively. The mean percentage of recovery for PCB congeners added to eight hair matrix spike samples was 91 ± 39%. The large SDs were a result of concentrations of some target analytes in hair being an order of magnitude higher than the amount of the spike added. Analytical precision, expressed as mean ± SD coefficient of variation for six triplicate and three duplicate hair samples, was 9 ± 8% for ∑PCBs, 9 ± 7% for *p*,*p*′-DDE (dichlorodiphenyldichloroethylene), 20 ± 9% for percentage lipid, and 7 ± 5% and 3 ± 3% for recoveries of two surrogates. The mean ± SD for ∑PCBs in procedural blanks for hair was 0.79 ± 0.08 ng. The average recoveries for two surrogates added to serum samples were 105 ± 6% and 93 ± 1%, respectively. The method detection limits (MDLs) for target analytes in serum ranged from 0.002 to 0.036 ng/g serum, with most MDLs < 0.01 ng/g serum (Korrick et al. 2000). The MDLs for hair samples ranged from 0.01 to 0.32 ng/g, with most MDLs < 0.1 ng/g. They were determined as 3 times the SD from the mean values for procedural blanks and using 0.5 g as the weight of the hair sample.

**Figure 1:**
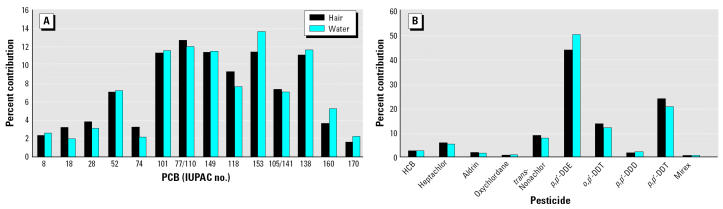
Contaminants in hair and washing liquid. (*A*) PCBs. (*B*) Pesticides.

The percentage of lipids and the levels of organic contaminants in hair were generally higher than those in serum. The levels in hair were above the detection limits for all compounds, except PCB-25 and dieldrin, which were not detected in hair; in serum, PCB-8, PCB-18, PCB-33, and PCB-37 concentrations were below the MDLs in all subjects, and PCB-26, PCB-44, and PCB-84 concentrations were below the MDLs in most of the subjects. Table 1 lists the median levels together with the 25th and 75th percentiles for organochlorines in hair separately for females, males, and all subjects. In a comparison of hair concentrations between hair washed with only hot water and hair washed once or twice with shampoo, washing hair with shampoo decreased the levels of PCBs, pesticides, and lipids by 25–33% on average (Table 2). For the less-chlorinated congeners, such as PCB-8 and PCB-18, this decrease was even larger, up to 48% and 62%, respectively. Most of the decrease in levels of organochlorines and the percentage of fat occurred after the first shampoo washing, with 82% of the total loss for ∑PCBs, 88% for *p*,*p*′-DDE, and 93% for percentage of fat. The percent contribution of each PCB congener to ∑PCB (or congener profile) in hair and washing liquid and the percent contribution of individual OCPs to their sum are presented in Figure 1.

**Figure 2:**
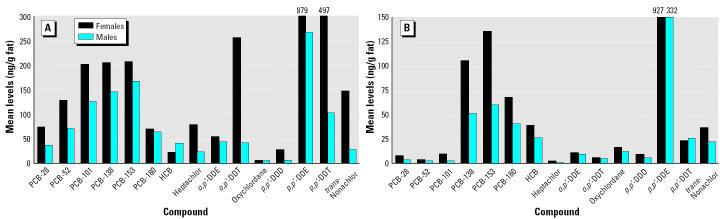
Mean levels (ng/g fat) of organochlorines in females versus males in (*A*) hair and (*B*) serum.

Two major contributors to the levels of organochlorines in serum are *p*,*p*′-DDE (47%), followed by the ∑PCB congeners (42%). Other contaminants contribute significantly less: 2% for *p*,*p*′-DDT, HCB, and *trans*-nonachlor and < 2% for other contaminants. There is a different percent distribution for the major contaminants in hair, with 70% of ∑PCB congeners, followed by only 14% of *p*,*p*′-DDE, and then 7% of *p*,*p*′-DDT. When the levels of OCPs in hair are compared between males and females, mean levels in females are significantly higher than in males (Figure 2A). The levels of pesticides, especially *p*,*p*′-DDE, in serum are also higher in females, although it is not as evident as for hair (Figure 2B).

**Figure 3:**
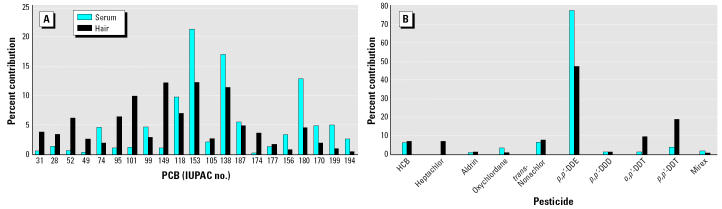
Contaminants in human serum and hair. (*A*) PCBs. (*B*) Pesticides.

PCB congener profiles for the average serum and hair concentrations are shown in Figure 3A, and the contribution of pesticides is presented in Figure 3B. The percentage of highly chlorinated and the more persistent PCBs were higher in serum than hair (Figure 3A). With the exception of PCB-74 (a persistent congener), the percentages of the less-chlorinated PCBs were higher in hair, for which a primary source may be from external exposure.

The mean ± SD ratios of *p*,*p*′-DDE: *p*,*p*′-DDT concentrations (nanograms per gram lipid) for all subjects were 28 ± 14 in serum versus 3 ± 2 in hair, which shows that DDE as a metabolization product is found in significantly higher proportions in serum than in hair.

The ratios and correlations of hair to serum concentrations for select PCB congeners and pesticides are shown in Table 3. A strong positive correlation was found between levels of *p*,*p*′-DDE in hair and blood, whereas moderate correlations were found for PCB-28, PCB-74, PCB-99, PCB-170, PCB-180, and PCB-194. A moderate negative correlation was found between levels of *o*,*p*′-DDE in hair and blood. The other PCB congeners and OCPs showed no correlation or weak correlation between the two matrices.

## Discussion

In the present study, we demonstrated that the analytical method for analyzing organic contaminants in human hair is both reliable and reproducible (coefficients of variation were < 10%). We found strong correlations (*r* = 0.8) between hair and blood levels of *p*,*p*′-DDE, the most stable metabolite of *p*,*p*′-DDT, which was abundant in both matrices. The correlation was stronger than the hair-to-blood correlation for *p*,*p*′-DDT, which was expected because *p*,*p*′-DDT is easily metabolized. A moderate hair-to-blood correlation was found for PCB-28, PCB-74, PCB-99, PCB-170, PCB-180, and PCB-194, which are the more persistent congeners. The other PCB congeners and OCPs showed no correlations or weak correlations between the two matrices. The negative correlation found between hair and blood levels of *o*,*p*′-DDE was unexpected. Although this may represent a chance finding, it is worthy of follow-up in future studies. We also explored correlations between hair and blood for the sum of easily metabolized congeners (e.g., PCB-31, PCB-52, PCB-101, PCB-110, PCB-132, and PCB-149), which are present in hair in higher proportions than in serum, but did not find strong correlations.

Several analytical methodologies for hair analysis have been previously described, each having advantages and disadvantages. A special emphasis has been placed on the determination of PCDDs/PCDFs and coplanar PCBs (Luksemburg et al. 2002; Nakao et al. 2002; Schramm et al. 1992), whereas major PCB congeners and pesticides (e.g., DDT) have been studied to a lesser extent (Covaci and Schepens 2001; Dauberschmidt and Wennig 1998). Several conclusions become evident after the evaluation of existing methodologies: *a*) the efficiency of extraction of organic pollutants from the hair matrix is enhanced after a chemical treatment (acid or base digestion) of the hair; *b*) liquid–liquid extraction of the hair digest is faster than the Soxhlet extraction and more efficient than solid-phase extraction procedures; *c*) because relatively low amounts of hair (< 1 g) are usually used, hair analysis can be miniaturized for lower solvent consumption, and the resulting cleaned hair extracts have less interfering compounds than do extracts obtained from other body matrices; and *d*) the use of digestion procedures, as well as the choice of adsorbents for extract cleanup, is strongly dependent on the analytes of interest. It has been shown that alkaline digestion destroys several OCPs (e.g., HCHs) and converts *p*,*p*′-DDT to *p*,*p*′-DDE and *p,p*′-DDD (dichlorodiphenyldichloroethane), whereas the use of acidified silica gel (33–44% concentrated sulfuric acid on silica) does not allow for the determination of acid-labile pesticides such as dieldrin and heptachloroepoxide (Covaci and Schepens 2001), present in measurable concentrations in other body matrices (i.e., serum and adipose tissue).

Compared with levels of PCB congeners with assigned World Health Organization toxic equivalency factors measured by Tirler et al. (2001) in hair samples from one person collected at three different time points (2 and 3 months apart), the concentrations in our subjects were similar for PCB-156, PCB-167, and PCB-189 but 2.5 times lower for HCB. Levels of PCB-105 and PCB-118 were 4 and 3 times higher, respectively, in our study. However, further comparison is not appropriate because Tirler et al. (2001) did not measure the most prevalent congeners, such as PCB-138, PCB-153, PCB-170, and PCB-180.

Nakao et al. (2002) measured levels of PCDD/PCDFs and coplanar PCBs in hair and blood collected from six healthy donors. The correlation factors between these two matrices for 1,2,3,4,7,8-hexaCDD and 2,3,4,7,8-pentaCDF were 0.63 and 0.93, respectively, whereas the other PCDD/PCDF isomers showed weak or no correlations. Both of these congeners were relatively abundant in the samples, especially 2,3,4,7,8-pentaCDF. With the exception of one sample, where 2,3,4,7,8-pentaCDF was not detected in blood, it was the most abundant PCDF congener (except for octaCDF) in blood. Also, the levels of this congener in blood were significantly higher than in hair, which indicates the persistence of this congener. There was also no correlation for the two tetrachlorobiphenyls, PCB-77 and PCB-81, which were not detected in blood in most of the samples. The levels of penta- and hexachlorobiphenyls, PCB-126 and PCB-169, in hair and blood were correlated, with correlations of 0.66 and 0.67, respectively. The levels of these congeners in blood were significantly higher than in hair, with the blood-to-hair ratios ranging from 5 to 24 for PCB-126 and from 40 to 190 for PCB-169. This limited information suggested that there was a correlation between hair and blood levels for more persistent compounds.

Environmental organic pollutants are deposited on and in human hair via two major routes, endogenous (dietary exposure followed by excretion of pollutants into the hair shaft) and exogenous (atmospheric deposition) (Schramm 1997, 1999). Therefore, hair reflects internal exposure to organic contaminants, as well as contamination from the environment and hair care products. Permanent hair treatments may also alter organochlorine levels in hair. Thus, the difficulty in separating externally deposited compounds from endogenously deposited compounds makes the interpretation of hair analysis difficult. Washing hair with soap and hot water should remove most externally bound contaminants and, theoretically, may allow for the determination of internally bound analytes. However, an additional factor is the endogenous excretion of organic pollutants through the sebaceous glands onto the hair shaft, which complicates the picture of exogenous versus endogenous exposure.

In the present study, we washed hair with hot water and shampoo in a covered vessel using a sonication bath. This adequately removed dirt and dust from the hair exterior. We did not wash hair with organic solvents because this can also remove endogenously bound contaminants from hair. The decrease in hair levels of both lipids and most organochlorine pollutants after washing with shampoo was similar, and it was mainly observed after the first wash (~ 25–35%), whereas additional (5%), although small, loss was observed after the second wash. The relatively low variations between the loss of these compounds during washing may be due to the imperfect structure of hair with various scratches and holes in the matrix, acting as adsorbing sites (Valkovic 1988), which results in the similar loss of pollutants during the washing, independent of their persistence in the human body. The decrease in the levels of pollutants after washing hair with shampoo is in agreement with results from Nakao et al. (2002), who showed that by washing hair with a common surfactant, levels of PCDDs and PCDFs in hair samples decreased by 50% and 64%, respectively, and that a second washing had no further effect on the elimination of PCDD/PCDFs from hair samples. Interestingly, the percent contribution of PCB congeners and OCPs to their sum in hair and washing liquid were similar (Figure 1). This fact, together with the decrease in the total levels of pollutants and lipids after the first shampoo wash, suggests that the washing procedure is probably able to remove lipidic material deriving from sebaceous excretion. This material has the same PCB profile (profile of washing liquid) as the PCB profile found in the inner side of the hair shaft (profile in hair after wash). This further suggests that the exogenous contamination is insignificant for most PCBs, including the persistent PCBs, but not for the very volatile congeners (e.g., PCB-8 and PCB-18). Therefore, the washing procedure with shampoo may be excluded, but a simple washing step with water is still needed, especially when animal hair (which might contain fine soil particles and other solid materials) is analyzed (Covaci A, unpublished data).

In the present study, the percentage of highly chlorinated and more persistent PCBs was higher in serum than hair and with the exception of PCB-74 (a persistent congener), the percentage of the less chlorinated PCBs was higher in hair. A primary source of the less chlorinated PCBs in hair may be from exogenous exposure from gaseous or particulate sources. Low-chlorinated PCBs have a higher vapor pressure and therefore are found in higher concentrations in air than are higher chlorinated PCBs (Vorhees et al. 1997). Furthermore, lower-chlorinated PCBs have a significantly shorter half-life time in the human body (due to a faster metabolization rate) and therefore are expected to contribute less to endogenous organochlorine exposure. The strong decrease (up to 62%) in the hair concentrations of the more volatile PCB congeners (e.g., PCB-8 and PCB-18) after shampoo wash supports the exogenous exposure hypothesis. However, more readily metabolized congeners, such as PCB-52, PCB-101, PCB-110, and PCB-149, are present at high concentrations in hair even after two washes with shampoo. This may be the result of different elimination and distribution mechanisms between hair and internal organs or body tissues.

The readily metabolizable compounds are found in lower concentrations in body organs and tissues, where they will accumulate only after passing through the liver and thus after being metabolized. The hair root is vascularized during its growth, and thus contaminants present in the blood stream may enter the hair shaft via the root. If the subject has had a recent exposure to a cocktail of contaminants (including the easily metabolizable ones) or if they have been continuously exposed at low or background concentrations, these compounds will be present in the blood stream for a limited time until they are metabolized. However, the compounds will be sequestered in the hair shaft and will be present in relatively higher concentrations than in serum. A similar mechanism may be valid for heptachlor, an easy metabolizable compound, which is present only in hair and not in serum (Figure 3B). On the other hand, oxychlordane, a metabolization product, is found in much higher amounts in serum than in hair (Figure 3B). The same hypothesis may apply to nonpersistent PCB congeners and *p*,*p*′-DDT, which have a much higher abundance in hair compared with serum. Significantly higher ratios of *p*,*p*′-DDE:*p*,*p*′-DDT concentrations in serum than in hair show that DDE as a metabolization product is found in higher proportions in serum than in hair. However, this is just a hypothesis, because there are no studies on the distribution of POPs between hair, blood, and other tissues.

Neuber and Merkel (1999) used hair samples from preschool children to assess indoor air pollution from lindane and DDT from wood preservatives, woodworking, or imported furniture in the homes from rural areas in Germany. They studied children because children’s hair was assumed not to be bleached or colored with hair agents. They did not wash hair samples before analysis and detected lindane in most of the samples and DDT in almost 30% of all samples (although the levels in most of the samples were below the quantification levels by gas chromatography–mass spectrometry). Their conclusion was that hair analysis is a suitable method for detecting and quantifying indoor air pollution by lindane and DDT, especially for screening purposes, because of its easy and noninvasive sampling. Tirler et al. (2001) suggested that hair can serve as a passive sampler, similar to spruce needles, and provide information on environmental exposures.

In summary, in the present study, we validated analytical methods for measuring PCBs and OCPs in human hair and evaluated the effect of hair washing on the levels of these contaminants. There were correlations between the levels in hair and blood for select organochlorine pollutants, including *p*,*p*′-DDE and more persistent PCB congeners, such as PCB-99, PCB-170, PCB-180, and PCB-194. However, because most organochlorines had a weak correlation or no correlation between two matrices, it is too early to recommend hair as a reliable biomarker of exposure to organochlorines, which can replace serum or tissues as a biomonitoring tool. The present study had a number of limitations, which included a small sample size, the lack of consistency in hair collection location on the scalp, and the variable time period between collecting hair and blood samples from the same individual.

Because there are several distinct advantages of hair analysis compared with blood or tissue analysis for organochlorine pollutants, further larger studies are recommended. Potential advantages of hair analysis include its utility in studies where it is not feasible to collect blood or tissue. Examples include large epidemiologic studies in which subjects can remotely collect their own hair and mail it to the investigator. In addition, hair analysis may be practical in studies on small children where it is not possible to collect blood samples. Finally, hair samples may also prove useful as a screening media to identify individuals or groups of individuals with high levels (e.g., special populations), ultimately allowing for more targeted and efficient studies using more traditional matrices, such as blood, breast milk, or adipose tissue.

## Figures and Tables

**Table 1:** Concentrations (ng/g fat) of select organochlorines and percent lipids in human hair, by percentile.

	Females (*n* = 5)			Males (*n* = 5)			All subjects (*n* = 10)		
PCBs (IUPAC nos.) and pesticides	25th	Median	75th	25th	Median	75th	25th	Median	75th
PCB congener									
6	5.4	12	33	13	18	60	11	15	39
8	15	18	53	27	41	43	18	34	53
16	7.9	18	51	6.4	17	27	8.0	18	32
18	14	45	103	8.6	21	31	11	24	51
26	18	27	52	17	17	17	17	22	39
28	26	37	136	22	35	50	22	36	53
31	24	51	144	26	30	35	24	32	60
33	18	34	119	29	30	42	27	32	65
37	24	24	24	5.1	41	122	14	33	81
41	14	14	14	6.0	41	51	10	27	46
44	26	85	136	35	40	68	29	54	85
49	13	42	93	15	20	22	13	21	42
52	40	130	165	45	59	96	40	77	130
60	13	21	87	16	18	21	13	20	23
66	46	127	137	39	48	63	39	56	137
70	41	146	188	31	59	72	38	65	146
74	17	48	70	5.2	6.0	17	6.0	17	48
77/110	114	253	316	76	106	158	76	136	253
84	34	94	157	44	58	76	34	67	112
87	39	104	108	26	35	56	26	47	104
95	50	116	144	39	79	92	46	85	144
97	24	81	104	21	24	46	21	35	81
99	18	71	108	14	14	27	14	23	71
101	102	207	214	63	115	157	63	136	214
105	36	49	75	17	22	23	17	25	49
118	62	172	218	44	57	83	44	72	172
135	35	44	50	30	36	36	30	36	50
136	17	31	34	11	37	41	11	33	39
138	184	245	268	71	163	213	71	198	245
141	38	84	86	13	91	99	15	85	99
146	29	36	37	10	25	29	10	29	37
149	145	216	229	65	224	234	65	220	234
151	59	70	78	53	92	154	59	78	87
153	208	238	254	74	189	236	74	222	254
156	8.9	19	20	4.7	6.7	8.0	5.1	8.5	19
167	4.0	7.3	9.2	2.2	3.4	3.5	2.2	3.8	7.3
170	15	32	47	16	29	29	15	29	40
171	11	16	18	7.0	16	18	7.0	16	18
174	32	62	71	25	70	79	25	66	79
177	16	31	38	11	31	34	11	31	37
180	37	76	106	37	70	71	37	71	106
183	21	37	41	13	41	46	13	39	46
187	54	90	93	32	87	104	32	88	104
189	0.4	0.6	1.3	0.9	0.9	1.0	0.5	0.9	1.2
196/203	10	14	27	6.0	14	15	6.0	14	16
199	10	13	24	7.0	12	13	7.0	13	18
∑PCBs	2,010	3,620	4,500	1,180	2,140	3,130	1,180	2,640	3,620
Pesticide									
HCB	17	20	27	30	32	41	20	28	32
Aldrin	12	12	12	4.0	15	27	4.0	12	27
Heptachlor	10	23	50	12	21	22	10	21	50
*o,p*′-DDE	22	47	88	31	35	62	22	41	76
*o,p*′-DDT	24	156	488	29	34	47	29	34	94
*p,p*′-DDD	4.6	25	50	1.7	3.4	6.7	1.7	5.6	25
*p*,*p*′-DDE	241	517	820	128	199	217	128	229	731
*p,p*′-DDT	51	466	802	53	67	158	51	113	466
*trans*-Nonachlor	27	125	139	24	35	36	24	36	125
Percent lipids	2.1	2.2	3.1	0.77	1.6	1.8	1.6	1.8	2.2

**Table 2:** Organochlorine concentrations in hair and their percentage of loss after washing hair with shampoo.

PCBs (IUPAC nos.) and pesticides	Hot water wash [mean (ng/g)]*^a^*	Shampoo once [mean (ng/g)]*^a^*	Loss after one shampoo (%)	Shampoo twice [mean (ng/g)]*^a^*	Additional loss after 2nd shampoo (%)
PCB congener					
8	0.65	0.33	49	0.34	0
18	0.39	0.21	47	0.15	16
16	0.59	0.45	24	0.40	8
26	1.3	1.2	11	1.1	1
31	1.6	1.2	21	1.1	7
28	1.2	0.92	25	0.81	9
33	0.92	0.82	11	0.75	8
52	3.6	3.1	13	2.9	7
49	1.5	1.1	25	0.93	11
44	2.2	1.9	13	1.9	3
95/66	7.2	5.6	22	5.3	4
74	1.6	1.1	30	1.0	6
70	3.8	3.3	14	3.2	2
84	4.1	4.0	2	3.7	8
60	0.77	0.58	25	0.47	14
99	3.7	2.6	28	2.4	8
101	6.5	4.8	26	4.5	4
97	2.7	2.0	26	1.8	6
87	3.1	2.4	22	2.3	4
77/110	6.0	5.1	15	5.6	7*^b^*
151	2.2	1.4	34	1.3	7
135	1.2	1.1	11	0.97	9
149	4.7	3.4	28	3.2	4
118	6.9	5.1	26	4.8	5
146	1.3	0.83	36	0.82	1
153	9.2	6.6	28	6.3	4
105/141	4.0	2.9	28	2.7	5
138	8.5	6.4	25	5.9	6
187	2.7	1.9	28	2.0	5*^b^*
183	1.2	0.83	30	0.82	1
128	0.62	0.44	29	0.37	12
174	2.3	1.7	27	1.6	5
167	0.34	0.24	29	0.22	5
177	1.2	0.89	27	0.83	5
157/201	0.44	0.34	22	0.30	8
171	0.57	0.43	24	0.39	7
156	0.86	0.60	30	0.51	11
180	3.6	2.6	28	2.4	5
170	1.8	1.3	27	1.2	5
199	0.82	0.57	31	0.52	6
196/203	0.89	0.65	27	0.60	5
189	0.06	0.04	21	0.04	8
195	0.28	0.22	22	0.20	5
194	0.40	0.29	29	0.26	5
∑PCBs	110	85	23	80	5
Pesticide					
*p,p*′-DDE	80	63	22	60	3
*o,p*′-DDE	3.0	2.4	21	2.2	6
*p,p*′-DDT	35	27	21	25	7
*o,p*′-DDT	22	17	24	15	6
*p*,*p*′-DDD	1.5	1.2	21	1.2	0
HCB	0.67	0.66	1	0.60	10
Heptachlor	0.61	0.51	15	0.51	1
*trans*-Nonachlor	4.3	3.1	27	2.7	9
Percent lipids	3.1	2.3	26	2.2	2

a Mean values for three replicate experiments.

b Increase.

**Table 3:** Ratios and correlations of hair to serum concentrations (ng/g fat) for select PCB congeners and pesticides.

	Hair:serum ratios (mean ± SD)			
PCBs (IUPAC nos.) and pesticides	Females (*n* = 4)*^a^*	Males (*n* = 5)	All subjects (*n* = 9)*^a^*	Spearman correlations
PCB congener				
28	6.5 ± 5.5	12 ± 5.6	9.0 ± 5.9	0.5
52	53 ± 78	49 ± 35	51 ± 54	−0.03
74	2.3 ± 2.9	1.3 ± 1.3	1.7 ± 2.1	0.5
99	3.6 ± 5.0	2.0 ± 1.3	2.7 ± 3.4	0.5
101	52 ± 82	48 ± 33	50 ± 55	0.2
149	47 ± 48	68 ± 52	59 ± 48	0.1
118	3.1 ± 3.5	3.5 ± 2.0	3.3 ± 2.6	0.4
153	1.8 ± 1.4	2.9 ± 1.5	2.4 ± 1.5	0.2
105	6.8 ± 7.4	12 ± 17	10 ± 13	0.2
138	2.5 ± 2.3	3.3 ± 1.8	2.9 ± 1.9	0.2
180	0.9 ± 0.5	1.7 ± 0.75	1.3 ± 0.75	0.6
170	1.0 ± 0.6	2.0 ± 1.1	1.6 ± 1.0	0.5
194	0.5 ± 0.5	0.9 ± 0.5	0.7 ± 0.5	0.6
∑PCBs	5.5 ± 6.5	6.3 ± 3.4	6.0 ± 4.7	0.2
Pesticide				
*o,p*′-DDE	6.7 ± 6.1	5.8 ± 4.2	6.2 ± 4.2	−0.6
*p*,*p*′-DDE	0.8 ± 0.7	0.9 ± 0.47	0.8 ± 0.47	0.8 *
*p*,*p*′-DDT	17 ± 17	7.9 ± 12.6	11.8 ± 12.6	0.4
*o,p*′-DDT	48 ± 53	9.0 ± 38.4	26.2 ± 38.4	0.4

a Percent lipids for one serum sample was not available.

* *p* < 0.05.
